# Correspondence regarding ‘Systematic review and meta-analysis of goal-directed haemodynamic therapy algorithms during surgery for the prevention of surgical site infection’

**DOI:** 10.1016/j.eclinm.2025.103236

**Published:** 2025-05-03

**Authors:** Hans Bahlmann, Ingvar Halldestam, Lena Nilsson

**Affiliations:** a Department of Anaesthesiology and Intensive Care in Linköping, Linköping University, 58185, Linköping, Sweden; b Department of Biomedical and Clinical Sciences, Linköping University, 58185, Linköping, Sweden; c Department of Surgery in Linköping, Linköping University, 58185, Linköping, Sweden

With great interest we read the recent meta-analysis on the effect of goal-directed haemodynamic therapy on surgical site infection by Jalazadeh et al.^1^ We were glad to find that a previous study of our group was included in the analysis.^2^ However, the data for the primary outcome as shown in Fig. 2 appear to differ from our original published data. The same applies to the secondary outcomes “reoperation” and “type of intervention 7E”, though in the latter case all included studies appear to be mis-classified, probably because the word “not” appears to be positioned in the wrong place.

Unfortunately this is not a new phenomenon to us. To our knowledge, our previous study published in 2019 has been included in five meta-analyses providing quantitative data on individual study level. In four of these five, in our opinion data differ from the original study for at least one parameter. This could of course be caused by random error or differing definitions, however in 9/11 cases (82%) of differing data, the error strengthens the conclusion of the new analysis (data available upon request). Some degree of systematic bias thus appears possible.

Performing clinical trials is hard work, and noticing that the results contribute to the progress of science by being correctly incorporated in a well-performed meta-analysis is a great reward. We therefore humbly call for additional measures to increase the reliability of the meta-analytic process.^3^ Perhaps one way would be to check with the original authors that they agree on the way their data are handled in the meta-analysis, wherever possible.

## Contributors

Hans Bahlmann conceptualised and wrote the original draft, which was reviewed and edited by Ingvar Halldestam and Lena Nilsson. All authors approved the final version of the manuscript.

## Declaration of interests

Hans Bahlmann has received a speaker fee from Baxter. Ingvar Halldestam and Lena Nilsson declare no conflicts of interest.
